# Fine-scale genetic structure analyses suggest further male than female dispersal in mountain gorillas

**DOI:** 10.1186/1472-6785-14-21

**Published:** 2014-07-07

**Authors:** Justin Roy, Maryke Gray, Tara Stoinski, Martha M Robbins, Linda Vigilant

**Affiliations:** 1 Max Planck Institute for Evolutionary Anthropology, Deutscher Platz 6, D-04103, Leipzig, Germany; 2 International Gorilla Conservation Program, P.O. Box 931, Kigali, Rwanda; 3 The Dian Fossey Gorilla Fund International and Zoo Atlanta, Atlanta, Georgia 30315, USA

**Keywords:** Dispersal, Inbreeding avoidance, Microsatellite, Mountain gorilla, Philopatry, Social mammal, Spatial genetic structure

## Abstract

**Background:**

Molecular studies in social mammals rarely compare the inferences gained from genetic analyses with field information, especially in the context of dispersal. In this study, we used genetic data to elucidate sex-specific dispersal dynamics in the Virunga Massif mountain gorilla population (*Gorilla beringei beringei*), a primate species characterized by routine male and female dispersal from stable mixed-sex social groups. Specifically, we conducted spatial genetic structure analyses for each sex and linked our genetically-based observations with some key demographic and behavioural data from this population.

**Results**

To investigate the spatial genetic structure of mountain gorillas, we analysed the genotypes of 193 mature individuals at 11 microsatellite loci by means of isolation-by-distance and spatial autocorrelation analyses. Although not all males and females disperse, female gorillas displayed an isolation-by-distance pattern among groups and a signal of dispersal at short distances from their natal group based on spatial autocorrelation analyses. In contrast, male genotypes were not correlated with spatial distance, thus suggesting a larger mean dispersal distance for males as compared to females. Both within sex and mixed-sex pairs were on average genetically more related within groups than among groups.

**Conclusions**

Our study provides evidence for an intersexual difference in dispersal distance in the mountain gorilla. Overall, it stresses the importance of investigating spatial genetic structure patterns on a sex-specific basis to better understand the dispersal dynamics of the species under investigation. It is currently poorly understood why some male and female gorillas disperse while others remain in the natal group. Our results on average relatedness within and across groups confirm that groups often contain close relatives. While inbreeding avoidance may play a role in driving female dispersal, we note that more detailed dyadic genetic analyses are needed to shed light on the role of inbreeding avoidance as an ultimate cause of female dispersal in mountain gorillas.

## Background

Social mammals have been arguably one of the most extensively studied groups of taxa over the last few decades and scientists have gathered valuable information spanning a large array of biological fields in many species [1-3]. For example, we know that most group-living mammals exhibit a polygynous mating system in which one dominant male usually monopolises reproduction with a number of adult females in the group [4]. A direct consequence of this non-random mating system is high variance in mating success among males, so that most offspring are sired by only one (or a few) adult male(s). From an evolutionary standpoint, the resulting presence of a large number of individuals that are closely genetically related to each other (hereafter termed kin) may have created social conditions from which kin selection developed; that is, individuals gain inclusive fitness benefits by enhancing the reproduction of their relatives [5]. Individuals remaining at or near their natal location may therefore benefit from the opportunity to interact with kin, but may also risk competition with kin or inbreeding (reviewed in [6]). Furthermore, evidence of inbreeding depression (i.e. decline in fitness of inbred progeny, [7]) has been reported for many groups of mammals (e.g. rodents [8,9], ungulates [10,11], and primates [12,13]). As a result, for an individual, there are incentives both to leave (dispersal) and to remain (philopatry) in its natal area. Importantly, these incentives need to be weighed against the costs of dispersal, which are likely to differ not only between males and females but also among same-sex individuals of a given species [14]. For that reason, dispersal patterns almost always differ between sexes and in most social mammals the males leave their natal group at a higher average frequency [15].

In addition to differences in dispersal frequency, it is also interesting to consider variation in dispersal distance among individuals of the same or different sexes [16]. Proper assessment of the extent of dispersal is an arduous task in mammals and feasible study areas may not encompass the actual dispersal distance achieved by individuals [17]. Thus, dispersal distance estimates derived from field studies may be highly biased, and even estimates derived from indirect genetic methods (e.g. F_ST_ ≈ 1/(4*N*_*e*_*m* +1), [18]) suffer from a lack of realism in the model assumptions. Over the last 10–15 years, following important developments in statistical methods applicable to the field of population genetics (e.g. multivariate spatial autocorrelation methods, [19]), there has been an increasing number of studies performing spatial genetic structure analyses using hypervariable genetic markers [20-22]. Spatial genetic structure analyses aim to detect non-random spatial distribution of genetic variation, ideally at different spatial scales, and are assumed to reflect the long-term effect of the reproductively effective dispersal of individuals [23]. Investigating sex-specific patterns of spatial genetic structure in social mammals at the levels of both within and among groups appears a promising avenue for shedding light on the evolutionary factors affecting dispersal in a given species (e.g. [24,25]).

**Figure 1:**
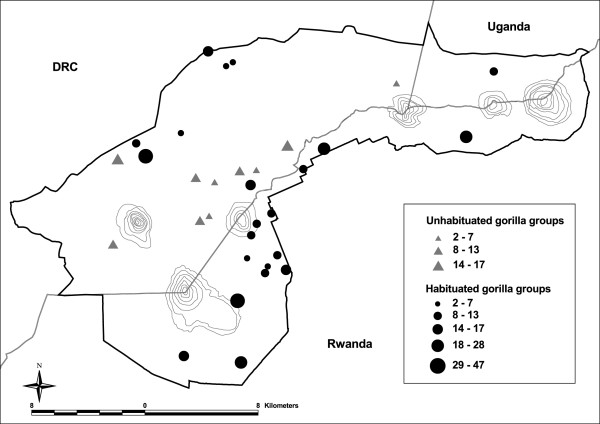
**Map of the Virunga Massif within the African continent.** The average GPS nesting locations of both habituated and unhabituated gorilla groups (*n* = 32 groups) used in this study are shown. Figure modified from Figure One in [56].

In this study, we use a set of 11 microsatellite genetic markers to investigate the spatial genetic structure of a primate species exhibiting routine male and female dispersal from stable mixed-sex social groups, the mountain gorilla (*Gorilla beringei beringei*). The study site is the Virunga Massif (Figure 1) and wholly encompasses the range of this population. We first describe and link our genetically-based observations with some key demographic and behavioural data from this population, which has been extensively monitored in the field over the last 45 years [26,27]. We then briefly discuss our results in terms of their implications on the role of inbreeding avoidance as an ultimate cause of female dispersal in the mountain gorilla.

The mountain gorilla is one of two eastern gorilla subspecies [28] and members of both sexes are known to disperse from their natal group around reproductive maturity (i.e. females: 8+ years old, silverback males: 12+ years old, [29]). Not all females however disperse before reaching reproductive maturity and these females often reproduce in their natal group [30]. In comparison with western lowland gorilla populations (*Gorilla gorilla gorilla*), mountain gorillas exhibit much lower rates of male dispersal and a correspondingly higher proportion of multi-male groups [31]. Assessing the fate of dispersing silverback males is a difficult task since they first enter a solitary phase, usually lasting for several years, before potentially forming and leading new groups [32]. Each group is led by a dominant silverback male who sires most or all of the offspring in the group, although copulations can also be performed by subordinate males in multi-male groups [33-35]. It has been suggested from analyses of genetic data from other gorilla populations that dispersing females may form kin associations within a group (western gorillas, [36]) and live spatially close to other female relatives (Bwindi mountain gorillas, [37]). Controversy still exists regarding males for which both the presence of dispersed male kin networks [38] and the absence of spatial genetic structure [37,39] have been reported. Based on these observations and the typical male-biased dispersal pattern in social mammals [6,15], we predict a spatial genetic structure for females but not males at the group level. Within a group, we expected mature same-sex individuals to be on average more genetically related to each other than to same-sex individuals of different groups.

## Results

### Individual identification

During the two-month sampling period, a total of 920 fecal samples were collected. Roughly one-third (307) of these originated from the unhabituated groups. We extracted DNA from a total of 480 samples, namely 236 and 244 samples originating from unhabituated and habituated groups, respectively. We used proportionally fewer of the habituated gorilla samples since their identity (and genotype) was known with certainty for most of them. We attempted genotyping from the 452 extracts (94.2%) that yielded positive results in the amelogenin sexing assay. Of these, 395 extracts produced genotypes at six or more loci. When using the software CERVUS to identify potential replicate samples of an individual, we obtained a low number of pairs of potential replicates matching at a minimum of six loci but mismatching at up to two loci (n = 25 pairs, out of 32 385 possible pairs). After considering information regarding dung size, date of nest site, group of residence and sex identification, and after combining genotypes perfectly matching at all loci, we were able to create a list of 255 unique individuals whose genotypes were on average 90.7% complete. A genotype is complete at a locus when both alleles are confirmed at this locus. Of these 255 unique individuals, 171 were classified as a silverback male or adult female based on field and genetic data. The genotypes from an additional 22 individuals from habituated groups sampled as part of another study [33] were added to our dataset, since they represented mature individuals known to indeed be part of such groups but not sampled during the 2010 census (i.e. individuals that were missed during the genetic census but known to be part of the groups at that time). Therefore, for the purpose of the genetic structure analyses, the number of mature individuals was 193, representing 75 silverback males and 118 adult females. For all 193 individuals, data were available for 11 microsatellite loci and we used the information at these loci to perform all subsequent genetic structure analyses.

### Standard genetic analyses

There were between four and seven alleles per locus, with an average of 5.55 (Table 1). Observed heterozygosity values ranged between 0.484 and 0.676 per locus, while expected heterozygosity values ranged between 0.492 and 0.707 per locus. *F*_IS_ values at each locus were all nonsignificant (α = 0.05) after the Bonferroni correction factor was applied. Global *F*_IS_ value was also not significant (*P* = 0.2200). Seventeen pairs of loci deviated significantly (α = 0.05) from linkage equilibrium, a higher proportion (17/55 = 0.3091) than what would be expected by chance alone. As these deviations are very likely to be the result of the significant spatial genetic structure detected in the subsequent analyses (see sections below), all loci were assumed to be statistically independent and were retained for further analyses. Null alleles were suggested to be present at two loci (D14s306 and D6s474), so we manually eliminated all apparent homozygotes at both loci by replacing one allele by a missing value. For all subsequent genetic structure analyses, we used the same 11 loci for all individuals.

**Table 1:** Summary of the genetic variation characteristics of the 11 microsatellite loci used in this study

**Locus**	**No. alleles**	**Pl**_**sib**_	***H***_**O**_	***H***_**E**_	***F***_**IS**_
D14s306	4	0.5079	0.535	0.603	0.113
D16s2624	5	0.4775	0.651	0.641	-0.016
D1s550	5	0.4745	0.613	0.649	0.056
D2s1326	6	0.4888	0.609	0.625	0.027
D4s1627	6	0.4818	0.601	0.630	0.046
D5s1470	7	0.4326	0.676	0.707	0.043
D6s1056	5	0.5377	0.526	0.550	0.044
D6s474	5	0.4487	0.607	0.680	0.108
D7s817	6	0.5471	0.508	0.536	0.052
D8s1106	7	0.5838	0.484	0.492	0.016
vWf	5	0.5294	0.544	0.578	0.058
Overall		4.784×10^-4^	0.578	0.608	

Genetic data was obtained from the entire sample of 255 individuals. Pl_sib_ is the probability of the identity among siblings; *H*_o_ is the observed heterozygosity; *H*_E_ is the expected heterozygosity; *F*_IS_ is the inbreeding coefficient. No. alleles denotes the number of alleles observed at a given locus. *F*_IS_ values are all nonsignificant (α = 0.05,10,000 permutations) after the Bonferroni correction factor is applied.

### Group-based genetic structure

Analyses of patterns of genetic variation used data only from samples known or estimated (based on dung size) to be from fully mature individuals living in groups (namely adult females and silverback males). Analyses of both sexes incorporated information from 32 groups (number of individuals per group = 2 – 17, mean ± SD = 5.63 ± 3.14, *n* = 180), for analyses of adult females we used 25 groups (number of females per group = 2 – 11, mean ± SD = 4.36 ± 2.34, *n* = 109), and for analyses of silverback males we used 17 groups (number of silverbacks per group = 2 – 6, mean ± SD = 2.88 ± 1.11, *n* = 49). Only groups containing two or more individuals of the same category were used for the analyses, and results did not differ when using only groups with four or more individuals per category.

**Figure 2:**
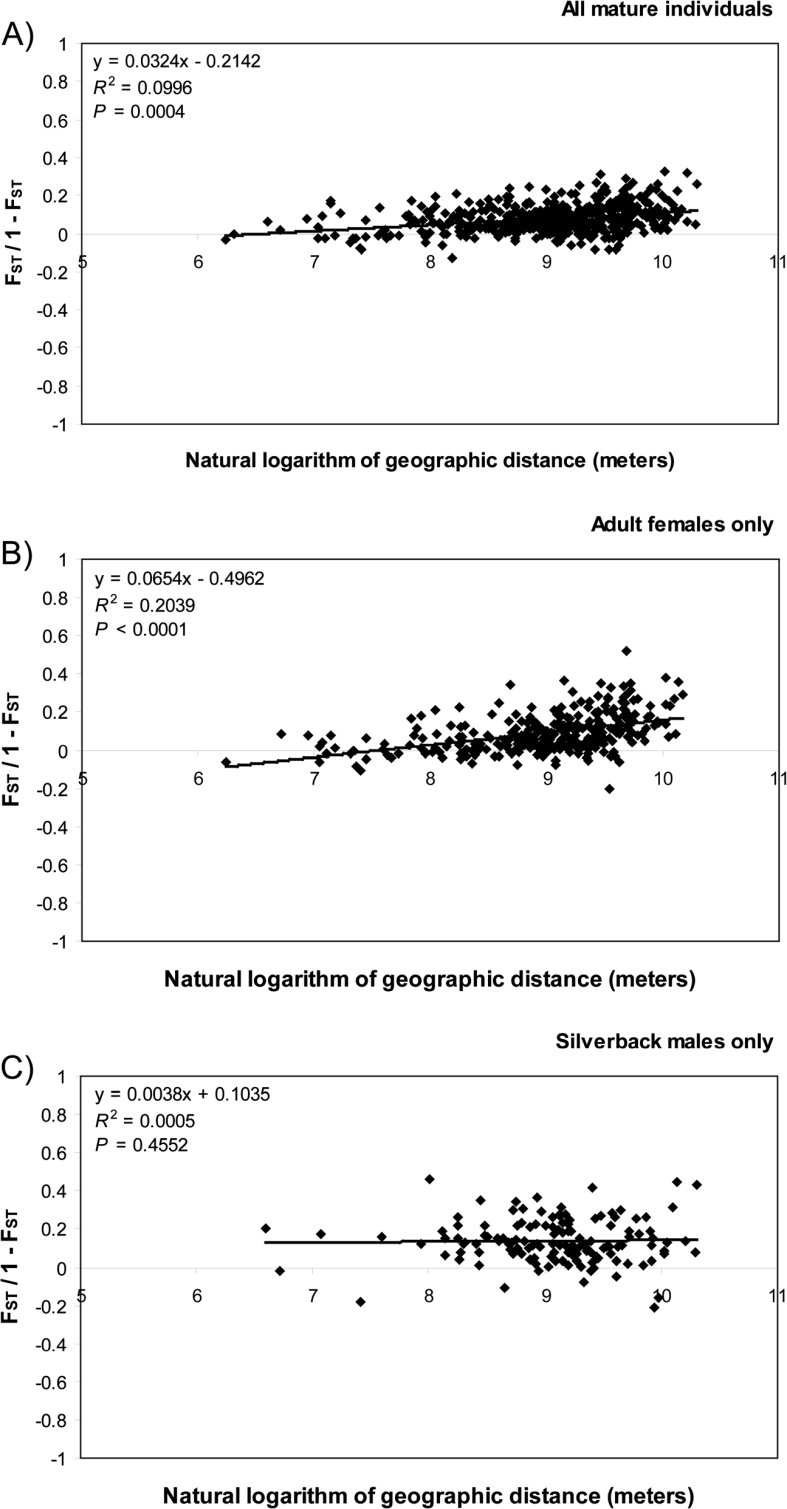
**Linear regression between pairwise F**_**ST**_**/(1 – F**_**ST**_**) ratios and the natural logarithm of geographic distance separating two groups.** Only groups containing two or more individuals were used in the analyses. **(A)** All mature individuals (*n* = 32 groups); **(B)** Adult females only (*n* = 25 groups); **(C)** Silverback males only (*n* = 17 groups). Regression lines are displayed for each plot. The regression equation, the coefficient of determination (*R*^2^), and the probability (*P*) to obtain a regression slope higher than the one observed are also reported. Note the identical scale for all plots.

Mantel tests revealed a significant and positive relationship between pairwise F_ST_/(1–F_ST_) ratios and ln-distance among groups for the comparisons involving all mature individuals as well as adult females only (*P* = 0.0004 and <0.0001 respectively, Figure 2A,B). Therefore, a pattern similar to an isolation-by-distance scenario was suggested by both of these comparisons. For this reason, it was inappropriate to run any large-scale population genetic software like STRUCTURE [40] on the whole data set since the underlying model is not suited to such kinds of data. In contrast, the regression involving exclusively silverback males was nonsignificant (*P* = 0.4552, Figure 2C). No significant relationship was found for any comparison involving the difference in altitude among groups as the independent variable when regressed against pairwise F_ST_/(1–F_ST_) ratios (results not shown).

A significant difference between the average dyadic relatedness values calculated within and among groups was found in all three types of comparisons. For all mature individuals, the observed mean within-group value was 0.0960, as compared to the among-group mean value of −0.0151 (difference of *r*_*ij*_ values = 0.1111, *P* <0.0001). For adult females, these values were respectively 0.0989 and −0.0068 (difference of *r*_*ij*_ values = 0.1057, *P* <0.0001), while for silverback males these values were respectively 0.2075 and −0.0387 (difference of *r*_*ij*_ values = 0.2462, *P* = 0.0004). In other words, for both males and females, two mature individuals belonging to the same group were on average more genetically related to each other than two mature same-sex individuals living in different groups. When considering mixed-sex pairs of individuals, we found that the average dyadic relatedness value within groups was significantly higher than among groups (observed mean within-group value = 0.0940, observed mean among-group value = −0.0132, difference = 0.1073, *P* <0.0001).

### Individual-based genetic structure

**Figure 3:**
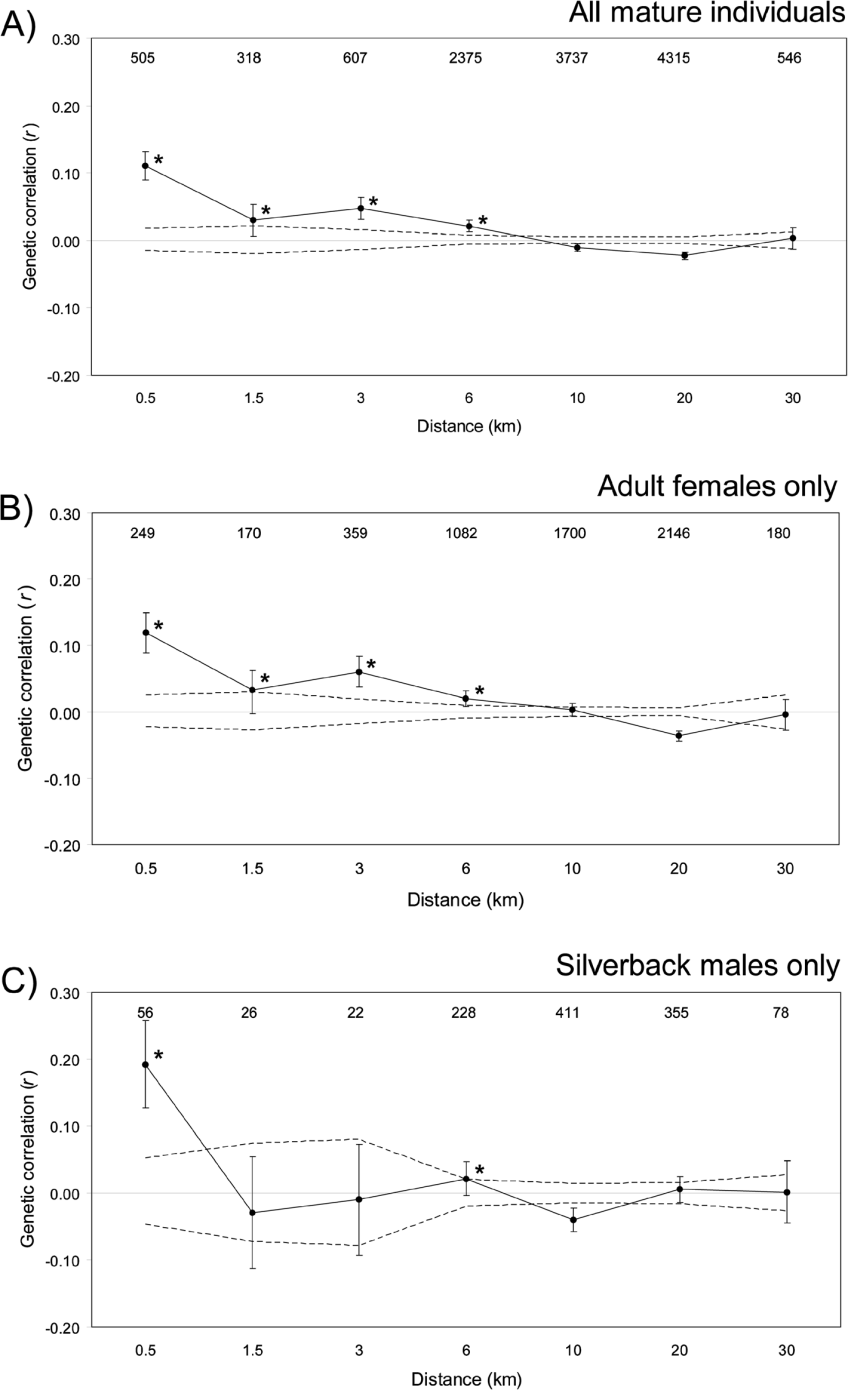
**Correlogram plots of the genetic correlation coefficient (*r*) as a function of geographic distance between individuals.** The 95% confidence interval about the null hypothesis of a random distribution of genotypes (dashed lines) and the bootstrapped 95% confidence error bars are also shown. The number of pairwise comparisons within each distance class is presented above the plotted values. **(A)** All mature individuals (*n* = 158); **(B)** Adult females only (*n* = 109); **(C)** Silverback males only (*n* = 49). All individuals of the same group fall within the 0.5 km distance class. Asterisks denote significantly positive *r* values at α = 0.05.

**Figure 4:**
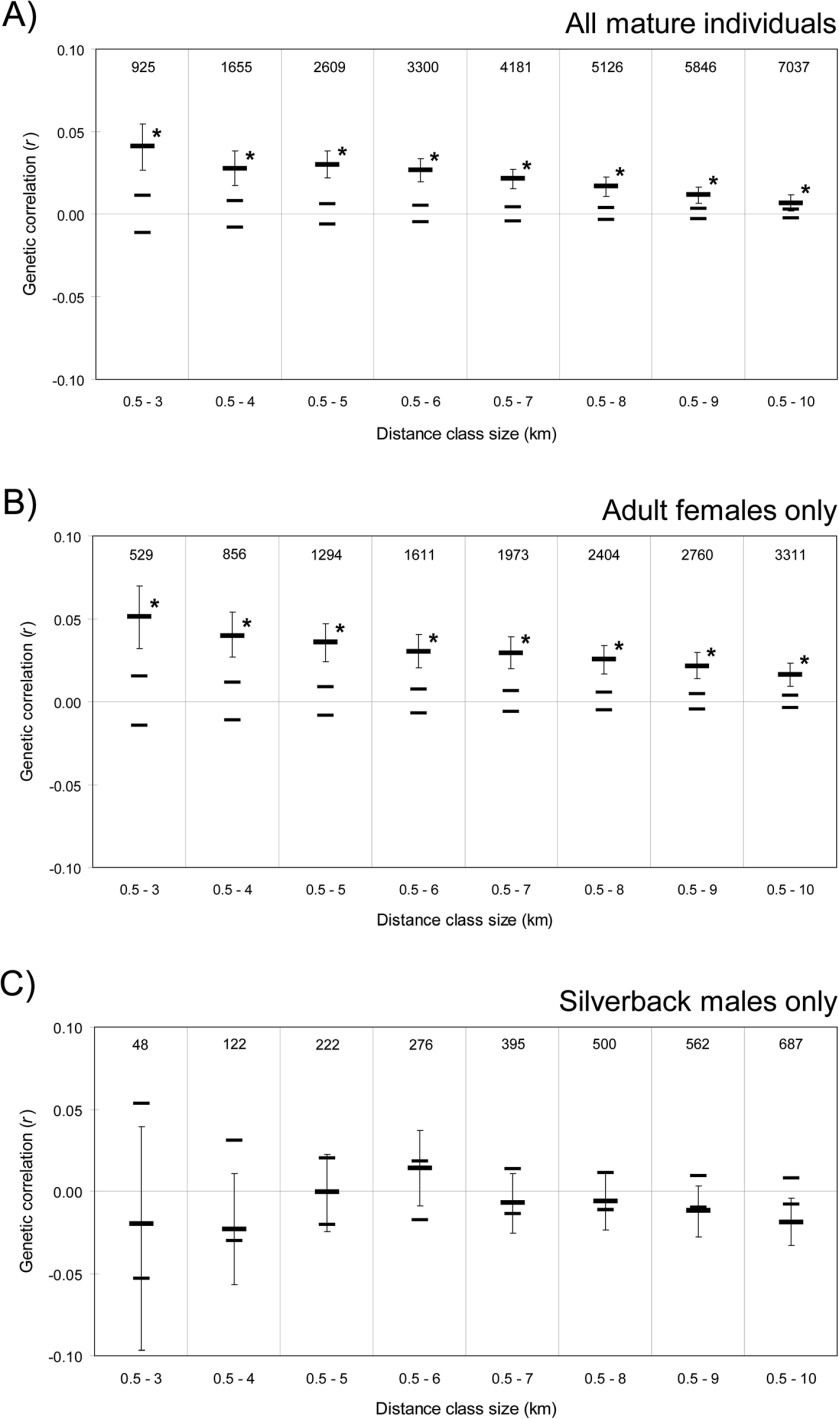
**Influence of modifying the second distance class on the spatial autocorrelation analyses.** Only the second distance class is shown, for increasing distance class sizes from 3 to 10 km. The thicker line denotes the genetic correlation coefficient (*r*), and the thinner lines indicate lower and upper bounds of the 95% confidence interval about the null hypothesis of a random distribution of genotypes. Bootstrapped 95% confidence error bars are also shown. The number of pairwise comparisons within each distance class size is presented above the plotted values. **(A)** All mature individuals (*n* = 158); **(B)** Adult females only (*n* = 109); **(C)** Silverback males only (*n* = 49). Asterisks denote *r* values significant at α = 0.05.

Spatial autocorrelation analysis of all mature individuals and adult females, respectively, revealed a positive and significant *r* value at the 1.5 km distance class (*P* = 0.0027 and 0.0126 respectively, Figure 3A,B), which was not the case for silverback males (*P* = 0.7930, Figure 3C) although there was a significant result at the 6 km distance class for the males, hinting at longer dispersal distance for males. Figure 4 illustrates the effect of successively increasing the size of the second distance class, from 3 to 10 km. Positive *r* values for all mature individuals as well as for adult females had a tendency to decline but remained significant beyond the distance class size of 10 km (Figure 4A,B). In contrast, analyses using silverback males did not reveal a significant positive autocorrelation at any of the distance class sizes (Figure 4C). Therefore, the interpretation of the results presented here is not dependent on the second distance class size defined.

### Observed female dispersal events

We analyzed a total of 92 female dispersal events occurring between 1995 and 2011 (*n* = 72 individuals). The magnitude of the movements varied from 0.51 to 8.34 km, with an average dispersal distance (±SE) of 3.18 km (±0.16). This value is similar to the mean shortest distance separating a group and its second nearest neighbouring group (mean ± SE: 2.73 ± 0.30 km, field data from 2010). The limited number of observed male dispersal events for which the fate of the male is known precludes a similar analysis in males.

## Discussion

The main objective of our study was to investigate the genetic signal of dispersal in the mountain gorilla population of the Virunga Massif by means of spatial genetic structure analyses. The results obtained here were largely consistent with our predictions, which were mainly based on results from field observations of dispersal patterns among the habituated groups of the Virunga mountain gorilla (*Gorilla beringei beringei*). First, the detection of population genetic structure at the group level was primarily driven by adult females, since a pattern of isolation-by-distance (IBD) was observed in females but not males. In addition, we found a positive genetic signal for females at the 1.5 km distance class in the spatial autocorrelation analyses. Therefore, adult females living in adjacent groups were on average more genetically related to each other compared to females living in more distant groups. This observation is in accord with observations that females during inter-group encounters almost exclusively transfer to neighbouring groups [41,42]. In support of this observation, we found that the mean distance of movements (mean ± SE: 3.18 ± 0.16 km, *n* = 92) achieved by 72 dispersing females between 1995–2011 in the Virunga Massif is similar to the mean shortest distance separating a group and its second nearest neighbouring group (mean ± SE: 2.73 ± 0.30 km, field data from 2010).

In contrast to the IBD pattern detected in females, the absence of genetic structure among adult males belonging to different groups is suggestive of a random spatial distribution of male genotypes within the whole area. The lack of a discernible genetic variation pattern in males was also observed in a study of the western lowland gorilla (*Gorilla gorilla gorilla*) in which males were also found to form a single undifferentiated population based on Y-chromosomal microsatellite markers [43]. However, another study of that same gorilla subspecies using autosomal microsatellite data suggested the presence of so-called “dispersed male networks”, namely a structure in which the single males leading multi-female groups had a tendency to settle closer to same-sex relatives after dispersal [38]. Our analyses were not designed to examine this scenario, which would require extensive investigation of dyadic genetic relationships among individual silverback males living in neighbouring groups (e.g. [38,39]). However, we note that several of the habituated groups formed after 2007 are led by males known from paternity analyses to be related [44]. Nonetheless, differences in sample size, ecological parameters such as population density, size of the area under analysis, type of genetic markers used and type of analyses performed, make comparisons between studies a challenging process. Among these factors, it is likely that the size of the study area under investigation plays an important role, although a detailed study looking specifically at various spatial scales is needed to confirm this hypothesis.

We suggest that the spatial genetic structure signal detected for females but not males at short distances likely reflects an intersexual difference in dispersal distance rather than dispersal rate. Female mountain gorillas appear to exhibit a stronger tendency to disperse from their natal group than in most other social polygynous mammals (e.g. domestic sheep [45], red deer [46], and white-tailed deer [47]), although a large proportion of female gorillas are known to be philopatric [48]. A recent study of groups in the Virunga Massif suggested that around 60% of 75 natal nulliparous females dispersed from their natal group before reproducing [48]. Likewise, in the core area of the Virunga Massif, about 47% of silverback males dispersed from their natal group [32]. Therefore, despite a similar dispersal rate among sexes, we observed different genetic patterns, which we argue is best explained by intersexual differences in dispersal distance rather than dispersal rate. Interestingly, the detection of female spatial genetic structure was possible despite the frequent occurrence of female secondary transfer in mountain gorillas (46% of females, [49,50]) and the possibility for females to transfer multiple times throughout their lives (e.g. up to 4 times in our analysis of movements).

A similar sex-specific genetic pattern was found in the other mountain gorilla population in Bwindi Impenetrable National Park, Uganda [37]. In that study, it was proposed that female dispersal is influenced by the distribution of gorilla food according to altitude. However, in contrast to that study for which geographic distances, altitude differences and changes in plant composition were each found to be significantly correlated with genetic distances for females, we found that among these factors only geographic distance is correlated with genetic distance in the Virunga mountain gorillas. Although we did not test for effects of plant composition on the genetic structure due to the lack of data in certain areas, this factor is not likely to be influential in the Virunga Massif since most group’s home ranges encompass multiple altitudinal and vegetation zones.

Another outcome of our analyses is the finding of significantly higher average dyadic relatedness values among adult members of the same group as opposed to that of members of different groups, independently of the sex considered. For females, this is in accordance with a finding in the western lowland gorilla that female kin associations could be present despite frequent natal and secondary dispersal events [36]. In that study, 40% of adult females had a female close relative in the same group, based on data from six groups. In social groups in which reproduction is dominated by a single male, as in the one-male groups of the western gorillas, females are presumed to emigrate to avoid inbreeding. In western gorillas, it has been proposed that females within a group could reach reproductive maturity at similar ages, thus allowing subsequently a potential “natal co-dispersal” of related female siblings [36] and this may also occur in the Virunga mountain gorillas. In addition, in the Virunga Massif, the high frequency of multi-male groups and reduced reproductive monopolization by the dominant male [33] may reduce the impetus for reproductive-age females to emigrate to avoid inbreeding, thus permitting the co-residence of natal female relatives [30]. Alternatively (but not exclusively), these female kin associations might have evolved due to social benefits such as higher frequency of affiliative behaviours, higher display of tolerance, and better support in conflicts among related female mountain gorillas than among unrelated ones [51,52]. In some other social mammals, associating with female kin was found to expedite the age of first reproduction (red howler monkey, [53]), reduce the length of inter-birth interval (white-faced capuchin, [54]), and increase weaning success (house mice, [55]). Finally, we cannot rule out the possibility that the significantly higher average dyadic relatedness values among adult females within a group might be due in part to the presence of some pre-dispersal females in the dataset. Indeed, it is very difficult to know from the field data alone whether a female is mature, since this status would only be confirmed when smaller dung belonging to another individual (most likely her infant) was found in the same nest. Only eleven such cases could be confirmed based on genetic data in the unhabituated groups, despite the high proportion (30-40%, [27]) of adult females in the whole population of gorillas and the fact that 75% of them ([56]) are assumed to have offspring at a given time.

For males, our relatively high and significant within-group mean relatedness value is consistent with an age-graded social system in which related adult males (mainly half-siblings, but also father-sons and full-siblings) are members of the same group for an extended period of time. This system could result from three main factors, namely the long breeding male tenure, the non-dispersal behaviour exhibited by some individuals and the high variance in reproductive success among silverback males. First, it is worth mentioning the long breeding tenure displayed by dominant males in several monitored groups over the last two decades (Figure 1 in [32]), which has contributed to the relatively high stability of group composition observed during that time. Second, in a recent demographic and behavioural study conducted in the core area of the Virunga Massif, and in stark contrast to what has been observed in the western lowland gorilla, approximately half of silverback males were not seen to disperse from their natal group during the course of the 40-year study [32]. As a consequence of this male philopatric behaviour, a significant proportion of groups (61% of habituated groups in 2010, [56]) contains silverback males that are presumably first or second degree genetic relatives [57]. The advantages for subordinate silverback males in adopting this reproductive strategy are numerous and include queuing for dominance status [58], performing a significant proportion of copulations with females in the group (ca. 40-55%, [35,59]) and thereby achieving occasional reproductive success [33], and potentially an average lifetime reproductive success higher than that of dispersing males [58]. Lastly, higher genetic similarity among males of the same group was also facilitated in our study area by the fact that the dominant male sires on average 85% of offspring in the group, as revealed by previous genetic paternity analyses [33]. The existence of kin groups of philopatric males is not the norm in mammals, but it has been reported in a few other species where females routinely disperse before reproduction, such as the Ethiopian wolf [60], chimpanzee [61] and hamadryas baboon [62].

The proximate and ultimate causes of male dispersal in mountain gorillas remain puzzling. Remaining in the natal group would seem to be the best strategy, as only about one-third of dispersing males were observed to form reproductively successful groups [57]. Models show that males should remain philopatric even under a wide range of conditions such as the presence in the natal group of multiple males and reproductive monopolization by the dominant [58]. Nonetheless, approximately half of monitored subordinate silverback males in the Virunga Massif dispersed from their natal group [32]. These dispersal events were characterized as voluntary, as they were not correlated with changes in rates of affiliation or aggression with the dominant silverback, and also appeared unrelated to myriad other factors such as the number of females, the group sex-ratio, or age of dominant silverback [58]. The lack of clear advantages to male dispersal suggests that males may disperse despite unfavorable fitness consequences and raises the intriguing possibility that male life history strategies in mountain gorillas are not yet consistent with the social dynamics that would be optimal under the extreme ecological conditions experienced by mountain gorillas, who live at a higher altitude and with less fruit resources than all other gorillas [58].

The ultimate cause of natal dispersal for females has long been a subject of discussion [30]. Based on the observation that all females disperse from one-male groups before reproducing while some of them stay when an additional male is present in the group [51], Harcourt & Stewart [49] suggested that inbreeding avoidance is the main factor triggering female natal dispersal in mountain gorillas, as proposed previously for other great ape species [12,63]. A similar conclusion was reached by Clutton-Brock [1] in a meta-analysis of data collected from several polygynous mammal species, in which he showed that females usually transfer to other groups before first breeding when there is a chance that the resident males at the time of first conception are their father or other close relatives. This hypothesis also received support in a recent paper contrasting female dispersal strategies across 47 plural-breeding mammal species [64]. In contrast, Robbins *et al*. [30] did not find much support for the inbreeding avoidance hypothesis in the Virunga mountain gorilla population, although most nulliparous females were residing in multi-male groups and thus had potential mates that were not their father. The authors instead hypothesized that infanticide avoidance could be the ultimate cause of dispersal by natal nulliparous females, relying on the observation that these females are more likely to leave one-male groups [65], but recent analyses showed that the rates of infanticide and infant mortality do not vary between one-male and multi-male groups [48]. Our genetic study, by showing significantly higher average relatedness between co-residing males and females, provides results consistent with the idea that females may disperse in order to minimize close inbreeding with highly related males (e.g. father or son). However, we note the possibility that some females might disperse to groups whose dominant male is a close relative (e.g. brother or paternal half-sibling), thus raising the question of the limits of kin recognition. Further genetic data and dyadic analyses are required to better assess the relationship between the dispersal distance and the probability of mating with unrelated individuals, and ultimately to better infer the relative importance of inbreeding avoidance as an incentive for female dispersal in mountain gorillas.

## Conclusions

Our isolation-by-distance and spatial genetic structure analyses, performed on a sex-specific basis, provide evidence for an intersexual difference in dispersal distance as the main factor describing the genetic structure patterns observed in the mountain gorilla. Whilst the ultimate cause of dispersal for males remains to be elucidated, we argue that females might disperse in order to decrease the probability of mating with a highly genetically related male. We note, however, that the risk of close inbreeding might still exist despite female dispersal. Future studies aiming to shed light on the evolutionary causes of dispersal in mammal species should consider performing a close examination of dyadic relationships in addition to group- and individual-level analyses.

## Methods

### Study site

The study site is the Virunga Massif, an afro-montane forested area of ca. 450 km^2^ which spans the borders of eastern Democratic Republic of Congo, northwestern Rwanda and southwestern Uganda (Figure 1). It ranges in altitude between 1850 and 4507 meters above sea level, with the higher altitudes associated with the peaks of six extinct volcanoes (listed from west to east): Mikeno (4437 m), Karisimbi (4506 m), Bisoke (3711 m), Sabyinyo (3534 m), Mgahinga (3474 m) and Muhabura (4127 m). The areas above approximately 3600 m altitude were not surveyed since gorillas are not known to build their nests in them. Although the habitat consists of different vegetation zones, it offers abundant and evenly distributed food to gorillas throughout the year and this explains why gorilla groups spread over a large range of altitudinal levels.

### Density, spatial distribution and temporal stability of the gorilla groups

The average gorilla density in the whole region is ca. 1 individual/km^2^, although major local differences do exist [56]. In particular, the central southern area known as the Karisoke sector holds one of the highest densities of groups and individuals (3 individuals/km^2^) in the area [26,27]. The majority of the groups located in this area is habituated to human presence and is part of either research or tourist-oriented groups. In contrast, the northern, western and eastern parts of the Virunga Massif contain a low number of gorilla groups and the density of gorillas there is far less than in the central area of the park. Using GPS data from the 2010 genetic census, the shortest spatial distance between any two groups ranges between 0.22 and 5.85 km (mean ± SD: 1.91 ± 1.44 km). As a point of comparison, the same type of distance calculated using data for the year 2003 ranges between 0.18 and 5.67 km (mean ± SD: 2.02 ± 1.35 km), suggesting that shortest distances between groups are rather similar throughout time. Furthermore, we do not expect large movements for a given group in the habitat over the course of a few years, based on the small annual group home range sizes that characterize this population (3–15 km^2^, [29]).

Roughly 70% of the whole population is habituated to human presence and monitored on a daily basis. The group composition of the habituated groups has been relatively stable over the last decade, with the exception of four fission events taking place between 2007 and 2010 along with three new groups formed by habituated solitary males during the same time period [56]. When fission events occur, the resulting two groups live in close proximity from one another and their group composition is usually not altered significantly. On the other hand, it is not known whether the group composition of the unhabituated groups has been stable over the last decade since these groups are not monitored on a yearly basis. However, the fact that the number of unhabituated groups found in 2010 is the same as in 2003 (n = 12 groups, [56]), combined with little changes in the group composition of habituated groups, suggests that their group composition has witnessed little changes over this time period.

### Sample collection

Fecal samples were collected during a genetic census conducted in March and April 2010, according to the sweep method described previously for monitoring the mountain gorilla populations [56,66,67]. Six teams traversed the whole Virunga Massif systematically from west to east, looking for the presence of gorilla night nests. When nests containing dung less than five days old were encountered, nests were counted and the dung size was measured to allow the assessment of the age and sex composition of the group [67]. The following dung size categories were used: adult male (silverback, SB), > 7.2 cm (with silver hairs); adult female (ADF), 5.5-7.2 cm (along with smaller dung potentially originating from an infant); adult female or blackback male (MED), 5.5-7.2 cm; juvenile/subadult (JUV), <5.5 cm (individual nest); infant (INF), generally <4.0 cm (found in mother’s nest). Approximately 5 g fecal samples were collected and stored using the two-step protocol of temporary storage in an excess of ethanol followed by dessication using silica gel beads [68]. As many as three nesting sites per putative group were looked at in order to assess the group’s identity and composition, because not all nests may be detected at one site, some samples may fail to yield DNA, and individuals may build more than one nest per night. GPS locations and altitudinal data were recorded at each nesting site found during the sample collection.

### DNA extraction and microsatellite analysis

DNA extraction from fecal samples was carried out using the QIAamp DNA Stool Kit (QIAGEN) with slight modifications [68]. For the unhabituated groups, we used the field nest count data to identify, for each putative group, the nest site with the highest estimated number of individuals, and then extracted DNA from samples from that nest site. In order to confirm that these unhabituated groups were consistently identified, we also extracted DNA from a minimum of three samples from each of the other nesting sites for each group. In contrast, for the habituated groups, samples were usually extracted from only one nesting site and corresponded to all samples thought to originate from sexually mature individuals (i.e. SB, ADF, MED). DNA quality of each extract was assessed by the amplification of a sex-specific region of the amelogenin locus [69], which was additionally used for the sex identification of the sample. Samples found originating from blackback males (initially coded as MED and genetically identified as a male), juveniles/subadults (JUV) and infants (INF) were not included in the subsequent genetic structure analyses since they represented pre-dispersal individuals and would have otherwise biased such analyses [37]. Indeed, it is known from previous gorilla studies (e.g. [33]) that lower-ranking blackback males have very limited reproductive opportunities within their natal group and therefore should not be considered as adult males for the purpose of genetic analyses.

DNA extracts which successfully yielded a product in a test polymerase chain reaction (PCR) at the amelogenin locus were then amplified at 11 microsatellite loci using primers employed in a previous study [70]: D6s1056-D14s306 [71], and D1s550-D2s1326-D4s1627-D5s1470-D6s474-D7s817-D8s1106-D16s2624-vWf [72]. Amplifications were done using the two-step multiplexing approach (detailed in [70]). In the initial multiplexing step, all microsatellite loci were amplified in a single reaction containing a final volume of 20 μL: 2.0 μL of 10× reaction buffer, 1.4 μL of MgCl_2_ (25 mM), 1.0 μL of dNTP (2.5 mM), 0.8 μL of bovine serum albumin (BSA, 20 mg/mL), 0.96 μL of primer mix (3.125 mM for each primer), 0.1 μL of 0.5 U Super*Taq* (HT Biotechnology) premixed 2:1 with *Taq*Start Antibody (BD Biosciences), and 5 μL of template DNA. PCR thermocycling was performed in a PTC-200 thermocycler (MJ Research) and included an initial denaturation step of 9 min at 94°C, followed by 30 cycles of 20 s at 94°C, 30 s at 57°C and 30 s at 72°C, completed by a 4-min elongation step at 72°C. In the following singleplex step aiming to amplify each of the loci individually, 5 uL of 1:100 diluted multiplex PCR product was used as template, and all reactions were independently performed in a 20-μL reaction volume containing 2.0 μL of 10× reaction buffer, 0.7 μL of MgCl_2_ (25 mM), 1.0 μL of dNTP (2.5 mM), 0.8 μL of bovine serum albumin (BSA, 20 mg/mL), 0.5 μL of each forward (FAM-, HEX-, or NED-labelled) and reverse primer (10.0 mM for each primer), 0.08 μL of 0.5 U Super*Taq* (HT Biotechnology) premixed 2:1 with *Taq*Start antibody (BD Biosciences). The thermocycling conditions were as described above, except that primer-specific annealing temperatures were used for each singleplex PCR and varied from 55°C and 60°C (detailed in [70]). Up to four different PCR products were then pooled and electrophoresed on an ABI PRISM 3100 Genetic Analyser. Results were analysed with GeneMapper Software version 3.7 (Applied Biosystems) using GeneScan 400HD ROX-labelled as a size standard.

Three to four independent replicates of each extract were initially amplified in 96-well plates, and three to five negative controls (H_2_O) were used during the whole process. For all 11 microsatellite loci, an allele was recorded in the final (consensus) genotype only if it was seen in at least two independent positive PCRs. Up to nine additional replicate PCRs were performed to resolve any ambiguous genotypes. In another study of mountain gorillas using similar methodology, Guschanski *et al*. [73] found that three replicate PCRs per extract for the primers we used were sufficient to achieve 99% certainty that a homozygote is indeed such at a given locus. For this reason, an individual was assigned as homozygote at any microsatellite locus if the same allele was exclusively seen in at least three replicate PCRs. For sex identification, an individual was assigned as female if the 104-bp band was exclusively seen in four positive PCRs at the amelogenin locus, while the status of male was assigned if the 110-bp band was also seen in at least two positive PCRs. The entire genotyping process took place within a six-month period.

### Individual identification

In order to ultimately obtain a list of unique individuals of the whole population and a clear portrait of the composition of the groups, the program CERVUS 3.0.3 [74] was sequentially used for different purposes. In a preliminary step, we excluded from our dataset all extracts whose multilocus genotype was confirmed at five or fewer loci (i.e. extracts with higher vulnerability to genotyping errors), so as to analyze only extracts successfully genotyped at a minimum of six loci (out of 11). Since our first goal was to find all potential replicates of an individual among these extracts, we initially used CERVUS to identify sets of genotypes matching exactly at eight or more loci, without mismatching at any other locus. The eight-loci threshold was chosen since it yielded a high degree of discrimination among individuals, even in the rare cases where two samples could only be compared at the eight least informative loci (in this case, PI_sib_ = 5.193 × 10^−3^). We then combined these genotypes into a more complete consensus genotype after confirming the sex identification of the extracts. CERVUS 3.0.3 was launched a second time in order to identify any replicates of an individual that might be represented by multilocus genotypes differing at a few loci due to genotyping errors (i.e. allelic dropout and false alleles). To that end, genotypes matching at a minimum of six loci but mismatching at up to two loci were checked for data entry errors, since these genotypes are most likely to represent the same individual. We used information gathered from dung size, date of nest site, group of residence and sex identification to ultimately assess the possibility of these genotypes originating from the same individual. For instance, two nearly-matching genotypes obtained from samples collected in two distant areas in the park at similar dates would be assigned as different individuals. After performing all CERVUS analyses, we were able to create a list of unique individuals and detail the composition of the different gorilla groups identified in the census.

### Standard genetic analyses

CERVUS 3.0.3 [74] provided the following locus-specific information when all individuals were considered in the analyses: number of alleles, observed heterozygosity, expected heterozygosity, and the probability of identity among siblings. The program GENEPOP 4.1 [75] was used to calculate *F*_IS_ values [76], and to test for departure from Hardy-Weinberg equilibrium on a per-locus basis as well as for linkage disequilibrium between pairs of loci (α = 0.05). Markov chain parameters were set at 10,000 dememorizations, 1,000 batches and 10,000 iterations. The software MICRO-CHECKER [77] was applied to test for the presence of null alleles at each locus.

### Group-based genetic structure

Spatial genetic structure at the group level was examined in the Virunga Massif using Mantel tests. For the first two approaches described in this section, the comparisons were made for all mature individuals (i.e. adult females and silverback males), adult females only and silverback males only. Solitary silverback males (*n* = 9) were excluded from the analyses because here we focus on individuals living in stable breeding groups. Likewise, only groups containing two or more individuals of the same category (i.e. all mature, adult females only, silverback males only) were used in the analyses, which explains why the number of groups differs for each category. For all analyses described hereafter in this section, the Euclidean distance between any two groups (n_total_ = 32 groups) was calculated from GPS data collected during the 2010 genetic census, and corresponded to the difference between the arithmetic mean of the GPS nesting locations of each group identified in the census.

As a first approach, pairwise F_ST_/(1–F_ST_) ratios among groups were linearly regressed on the natural logarithm of geographic distance. Under an isolation-by-distance scenario, F_ST_/(1–F_ST_) is expected to vary linearly with the logarithm of the distance in a two-dimensional space [78]. The coefficient of determination (*R*^2^) was calculated for each regression using the program SPAGEDI 1.3d [79]. Elements of the group locations matrix were permuted 20 000 times (cf. Mantel test) to test for the significance of the observed regression slope (α = 0.05). *P*-value is reported as the probability to obtain by chance a regression slope higher than the one observed. For all comparisons, the null hypothesis of no spatial genetic structure was tested against the alternative hypothesis of spatial genetic structure expected under isolation-by-distance. Linear regressions and Mantel tests were also performed between pairwise F_ST_/(1–F_ST_) ratios and the difference in altitude among groups to assess the potential influence of this factor on the genetic distribution patterns.

As a second approach, the average dyadic relatedness values (*r*_*ij*_, following [80]) calculated within and among groups were compared against each other using a Microsoft Excel Macro developed by D. Lukas (available upon request). This Excel Macro uses, for each category tested, the set of individuals for which the relatedness values are calculated to estimate the reference allele frequencies. Queller & Goodnight’s coefficient (*r*_*ij*_) was chosen since it is independent of Hardy-Weinberg equilibrium conditions. The significance of the difference between both values was assessed by performing 10,000 permutations of the individuals among groups, but always keeping constant the number and the size of the groups. *P*-value is reported as the probability to obtain by chance a difference between these two values higher than the one observed.

As a last approach, we first calculated the average dyadic relatedness values (*r*_*ij*_, following [80]) of mixed-sex pairs of mature individuals within and among groups. The whole dataset of mature individuals was used to estimate the reference allele frequencies. We then calculated the observed absolute difference between these two values and assessed its significance (two-tailed test) by performing 10,000 permutations of the individuals among groups. *P*-value is reported as the probability to obtain by chance an absolute difference which is higher than the one observed. All these steps were performed in the software R [81].

### Individual-based genetic structure

In order to obtain a detailed picture of the relationship between pairwise genetic relatedness and geographic distance, particularly at small spatial scales, fine-scale genetic structure was further investigated using a global spatial autocorrelation technique implemented in the software GENALEX 6 [82]. The technique has been described in detail in the literature [19,83], its efficiency to detect sex-biased dispersal demonstrated by simulations [84], and the method increasingly used over the last few years [85-87]. In such analyses, the autocorrelation coefficient (*r*) is calculated for a number of pre-defined distance classes, and then compared to *r* values obtained under a random spatial distribution of individuals to test for its significance.

As for the group-based genetic structure analyses, the comparisons were made for all mature individuals, adult females only and silverback males only. For each comparison, the upper value of each distance class was first defined as a trade-off between the spatial resolution and the number of pairs of individuals in each class: 0.5 km, 1.5 km, 3 km, 6 km, 10 km, 20 km and 30 km. The first distance class (i.e. [0–0.5] km) contained exclusively comparisons among individuals of the same breeding group, whilst the upper limit of the second distance class (1.5 km) approximates the mean shortest distance between two adjacent groups (mean ± SD = 1.91 ± 1.44 km). Therefore, the main interest here resides in the detection of positive spatial autocorrelation in the second distance class (i.e. [0.5–1.5] km), as expected under dispersal to the nearest group. Given that the capacity of detecting spatial genetic structure is influenced in part by the defined distance class sizes [20], the same analyses were then performed but modifying only the second distance class as follows: every 1 km from 3 to 10 km, successively. Under a scenario of real positive spatial autocorrelation, the *r* value decreases but remains significant as the size of the second distance class increases. Results are presented as correlograms (plots of *r* as a function of distance), with 95% confidence intervals around *r* estimated by 1000 bootstraps. Positive spatial genetic structure was declared when the probability *P* to achieve by chance a value greater than or equal to the observed *r* was less than 0.05, as determined through 10,000 random permutations of the individual genotypes among the geographic locations.

### Observed female dispersal events

For comparison with the inferences made from genetic data, we assessed the extent of the known dispersal events made by females within the Virunga Massif. To that end, we used a long-term database recording the movements achieved by individuals living in habituated groups (both research and tourist-oriented groups) and focused on dispersal events taking place among those groups from 1995 through 2011. A dispersal event was here defined as any movement made by an individual from one group to another which was not the result of a group fission or fusion. We considered both natal and secondary dispersal. The limited number of observed male dispersals for which the fate of the male is known precludes a similar analysis in males.

Importantly, all cases in which a female was recorded as moving back and forth between two specific groups, independently of the total duration of it, were not considered as distinct movements and were counted at most once (dependent on the outcome). Likewise, if an individual initially moved from group A to group B and then from group B to group C, within a 1-month period, only the resulting distance (that is, between groups A and C) was considered in the analysis. For distances calculations, the GPS locations of gorilla groups for the years 2000, 2003, 2007 and 2010 were used. For any movement achieved by an individual, the date was known to within one week, and the calculation of the distance was performed using the closest year in time for which UTM coordinates were available. This approach seemed appropriate since we do not expect large movements for a given group over the course of a few years, especially considering the small annual group home range sizes that characterize this population (3–15 km^2^, [29]). The software GENALEX 6 [82] was used to calculate the shortest Euclidean distance between any group and its 10 nearest neighbouring groups.

## Competing interests

The authors declare that they have no competing interests.

## Authors’ contributions

JR carried out the experimental work of the study, did the analyses and wrote the manuscript. MG and MMR designed the sampling scheme, collected samples and gave advice on the manuscript. MG, MMR and TS participated in the recording of the demographic data used in this study. TS also gave advice on the manuscript. LV conceived of the study and wrote the manuscript. All authors read and approved the final manuscript.
